# Paratubular basement membrane insudative lesions predict renal prognosis in patients with type 2 diabetes and biopsy-proven diabetic nephropathy

**DOI:** 10.1371/journal.pone.0183190

**Published:** 2017-08-15

**Authors:** Koki Mise, Yutaka Yamaguchi, Junichi Hoshino, Toshiharu Ueno, Akinari Sekine, Keiichi Sumida, Masayuki Yamanouchi, Noriko Hayami, Tatsuya Suwabe, Rikako Hiramatsu, Eiko Hasegawa, Naoki Sawa, Takeshi Fujii, Shigeko Hara, Hitoshi Sugiyama, Hirofumi Makino, Jun Wada, Kenichi Ohashi, Kenmei Takaichi, Yoshifumi Ubara

**Affiliations:** 1 Nephrology Center, Toranomon Hospital Kajigaya, Kanagawa, Japan; 2 Okinaka Memorial Institute for Medical Research, Toranomon Hospital, Tokyo, Japan; 3 Department of Nephrology, Rheumatology, Endocrinology and Metabolism, Okayama University Graduate School of Medicine, Dentistry and Pharmaceutical Sciences, Okayama, Japan; 4 Yamaguchi’s Pathology Laboratory, Chiba, Japan; 5 Nephrology Center, Toranomon Hospital, Tokyo, Japan; 6 Department of Pathology, Toranomon Hospital, Tokyo, Japan; 7 Department of Human Resource Development of Dialysis Therapy for Kidney Disease, Okayama University Graduate School of Medicine, Dentistry and Pharmaceutical Sciences, Okayama, Japan; 8 Department of Pathology, Yokohama City University Graduate School of Medicine, Kanagawa, Japan; University of Louisville, UNITED STATES

## Abstract

**Aims:**

Glomerular insudative lesions are a pathological hallmark of diabetic nephropathy (DN). However, paratubular basement membrane insudative lesions (PTBMIL) have not attracted much attention, and the association between such lesions and the renal prognosis remains unclear.

**Methods:**

Among 142 patients with biopsy-proven DN and type 2 diabetes encountered from 1998 to 2011, 136 patients were enrolled in this study. Patients were classified into 3 groups (Group 1: mild, Group 2: moderate, Group 3: severe) according to the extent of cortical and medullary PTBMIL. The endpoint was a decline of the estimated glomerular filtration rate (eGFR) by ≥ 40% from baseline or commencement of dialysis for end-stage renal disease. The Cox proportional hazard model was employed to calculate hazard ratios (HRs) and 95% confidence interval (CIs) for the death-censored endpoint.

**Results:**

During a median follow-up period of 1.8 years (IQR: 0.9–3.5), the endpoint occurred in 104 patients. Baseline mean eGFR was 43.9 ± 22.8 ml/min/1.73 m^2^, and 125 patients (92%) had overt proteinuria. After adjusting for known indicators of DN progression, the HR for the endpoint was 2.32 (95% CI: 1.20–4.51) in PTBMIL Group 2 and 3.12 (1.48–6.58) in PTBMIL Group 3 versus PTBMIL Group 1. Furthermore, adding the PTBMIL Group to a multivariate model including known promoters of DN progression improved prediction of the endpoint (c-index increased by 0.02 [95% CI: 0.00–0.04]).

**Conclusions:**

PTBMIL may be useful for predicting the renal prognosis of patients with biopsy-proven DN, but further investigation of these lesions in various stages of DN is needed.

## Introduction

Glomerular insudative lesions are one of the characteristic histological features of diabetic nephropathy (DN). They are commonly known as capsular drops and fibrin caps, which are located between the parietal epithelium and Bowman’s capsule or within the glomerular capillary lumen, respectively [1,2]. Although the term ‘exudative lesion’ has long been used, insudative lesion is preferable because these lesions are localized ‘inside’ the lumen. The term fibrin cap is also poorly chosen because these lesions actually consist of plasma proteins with a homogeneous, glassy, and hyaline appearance, as well as lipid droplets, but do not contain fibrin [2,3].

Stout et al. reported that insudative lesions were not only observed within the glomerular capillaries and Bowman’s capsule, but also in the renal arteries and the proximal convoluted tubules [1]. The pathogenesis of such lesions is not well understood, although they speculated that hemodynamic factors may have a role and other researchers have suggested an association between the early development of these lesions and endothelial injury [1,4].

Recent studies of biopsy-proven DN have clearly demonstrated that glomerular and arteriolar insudative lesions are associated with the renal prognosis of DN patients, although the association is not as strong as interstitial fibrosis and tubular atrophy (IFTA) or nodular lesions [5–7]. On the other hand, the distribution and severity of proximal tubular insudative lesions remain unclear, as does the relationship of these lesions with the renal prognosis of DN patients.

In the present study, we focused on insudative lesions of the proximal tubules in patients with biopsy-proven DN, and assessed the extent of these lesions by assigning a histological score. We also investigated the association of insudative lesions with other characteristic histopathological features of DN and with the renal prognosis to determine the clinical utility of assessing these lesions of the proximal tubules.

## Materials and methods

### Study design

Among 142 patients with type 2 diabetes who underwent renal biopsy at Toranomon hospital from January 1998 to June 2011 and were confirmed to have isolated DN, 136 patients were eligible for enrollment in this study. The others were excluded because the estimated glomerular filtration rate (eGFR) was < 10 ml/min/1.73 m^2^ at the time of renal biopsy. DN was diagnosed by at least two renal pathologists and/or nephrologists, and the diagnosis was re-evaluated according to the classification of Renal Pathology Society (RPS) [2]. In this study, isolated DN was defined as DN without kidney transplantation or other coexisting renal diseases except nephrosclerosis. The protocol of this study was approved by the ethics committee of Toranomon Hospital in February 2015, and study procedures fully adhered to the Declaration of Helsinki. This study was registered with the University Hospital Medical Information Network (UMIN) in May 2016 (identification number: UMIN000022542). The medical records and other patient information were anonymized and de-identified prior to analysis.

### Laboratory parameters and definitions

GFR was estimated using the Chronic Kidney Disease Epidemiology Collaboration equation modified by the Japanese coefficient [8]. while baseline urinary protein excretion (UP) was measured in a 24-hour urine specimen. In this study, normoalbuminuria, microalbuminuria, and macroalbuminuria were respectively defined as urine albumin-to-creatinine ratio (UACR) <30 mg/gCr, UACR ≥30 and < 300 mg/gCr, and UACR ≥300 mg/gCr in at least two of three consecutive urine specimens obtained immediately before and after renal biopsy [9], while overt proteinuria was defined as macroalbuminuria or UP >1 g/day. hemoglobin A1c (HbA1c) data are presented as National Glycohemoglobin Standardization Program values according to the recommendations of the Japanese Diabetes Society and International Federation of Clinical Chemistry.[10] As in our previous studies, the average annual values of clinical parameters such as UP, systolic/diastolic blood pressure (BP), hemoglobin, and HbA1c were calculated [11–13]. Treatment with an angiotensin-converting enzyme inhibitor (ACE-I) or angiotensin II type I receptor blocker (ARB) during follow-up was defined as use by the patient for more than half of the follow-up period.

### Endpoint

The primary endpoint was defined as a decline of eGFR by at least 40% from baseline or commencement of dialysis due to end-stage renal disease (ESRD). We selected this outcome based on a recent meta-analysis of eGFR decrease in 1.7 million patients [14]. None of the patients received kidney transplantation during follow-up.

### Renal biopsy and pathological classification

The indications for renal biopsy were UP >0.5 g/day or atypical DN, such as nephritic syndrome with a short duration of diabetes or renal involvement without diabetic retinopathy and/or with hematuria, as described previously [5,15]. Tissue was obtained by needle biopsy and the specimens were processed for light microscopy, immunofluorescence, and electron microscopy. Specimens for light microscopy were stained with hematoxylin and eosin, periodic acid Schiff (PAS), Weigert’s elastica-van Gieson, Masson trichrome (MT), or periodic acid methenamine silver (PAM) stain according to routine methods. Biopsy specimens were also processed for immunofluorescence and electron microscopy in all patients for differentiation of other renal disease, as described previously [11,16]. DN was classified and histological scores were determined according to the criteria of the RPS and the criteria used in our previous study [2,5] by at least two renal pathologists and/ or nephrologists who were unaware of the clinical status of each patient.

We observed insudative lesions between tubular epithelial cells and the tubular basement membrane (TBM), and designated such insudative lesions as ‘paratubular basement membrane insudative lesions (PTBMIL)’ in this study. We also investigated the progression of PTBMIL from cortex to medulla using serial sections in some patients. PTBMIL was assessed by a single renal pathologist (Dr. Y.Y), and was classified into the following 4 categories separately in the cortical and medullary regions: grade 0, no PTBMIL; grade 1, PTBMIL in <25% of tubules in the region; grade 2, PTBMIL in 25%-50% of tubules, and grade 3, PTBMIL in >50% of tubules. The PTBMIL score (0–6) was calculated as the sum of the cortical and medullary PTBMIL grades, because this lesion develops from the proximal tubular pole through the proximal convoluted tubules in the cortex and extends to the proximal straight tubules in the medulla, which means that the severity of this lesion should be assessed by examining the medulla as well as the cortex. Thus, it should be noted that the IFTA score was mainly assessed in the cortex, whereas PTBMIL was evaluated in both the cortex and medulla. In addition, we classified the patients into three groups according to the PTBMIL score, which were PTBMIL group 1 (PTBMIL score of 0–2), PTBMIL group 2 (PTBMIL score of 3–4), and PTBMIL group 3 (PTBMIL score of 5–6), in order to develop a simple system for use in clinical practice.

In order to confirm the reliability and reproducibility of our PTBMIL scoring method, PAM/PAS/MT-stained slides from 30 patients were randomly selected and were independently evaluated by another renal pathologist (Dr. K.O). As a result, there was good inter-observer agreement between PTBMIL scores or PTBMIL groups evaluated by two renal pathologists (weighted κ value for PTBMIL score: 0.77, weighted κ value for PTBMIL group: 0.62) [17,18].

### Statistical analysis

Data were summarized as percentages or as the mean ± standard deviation [SD], as appropriate. Logarithmic transformation of skewed variables (UP, triglycerides, and total cholesterol) was done to improve normality before analysis. Categorical variables were analyzed with the chi-square test, Fisher’s exact test, and the two-group proportion test, while continuous variables were compared by using the paired t-test, Wilcoxon signed rank test, Mann-Whitney U test, Kruskal-Wallis H test, or ANOVA, as appropriate. The distribution of each clinical and histopathological parameter stratified by the PTBMIL group was compared using trend analysis. Correlations of the PTBMIL score/group and IFTA score with other pathologic findings were evaluated by Spearman’s correlation analysis. Cumulative renal survival was estimated for each PTBMIL group by the Kaplan-Meier method, and renal survival rates were compared among these groups by using the log-rank test. The Cox proportional hazards model was employed to calculate hazard ratios (HRs) and 95% confidence intervals (CIs) for the death-censored endpoint. In Cox model 1, HRs were adjusted for age, gender, body mass index (BMI), estimated duration of diabetes, diabetic retinopathy, and systolic BP at the time of renal biopsy. These covariates were selected as potential confounders on the basis of biological plausibility and metabolic memory [19,20]. In model 2, HRs were adjusted for all of the above covariates plus eGFR and log converted UP at the time of renal biopsy. In order to investigate the incremental predictive power of the PTBMIL group and PTBMIL score, we compared Harrell’s concordance index (c-index) between multivariate Cox proportional hazards models adjusted for the covariates in model 2 with or without the PTBMIL group and PTBMIL score. The 95% CIs for differences of the c-index were computed from 10000 bootstrap samples. Two-tailed P values < 0.05 were considered to indicate statistically significant differences. All analyses were performed with Stata SE software (version 14.0, StataCorp LP).

## Results

### Pathogenesis and progression of PTBMIL

**Fig 1:**
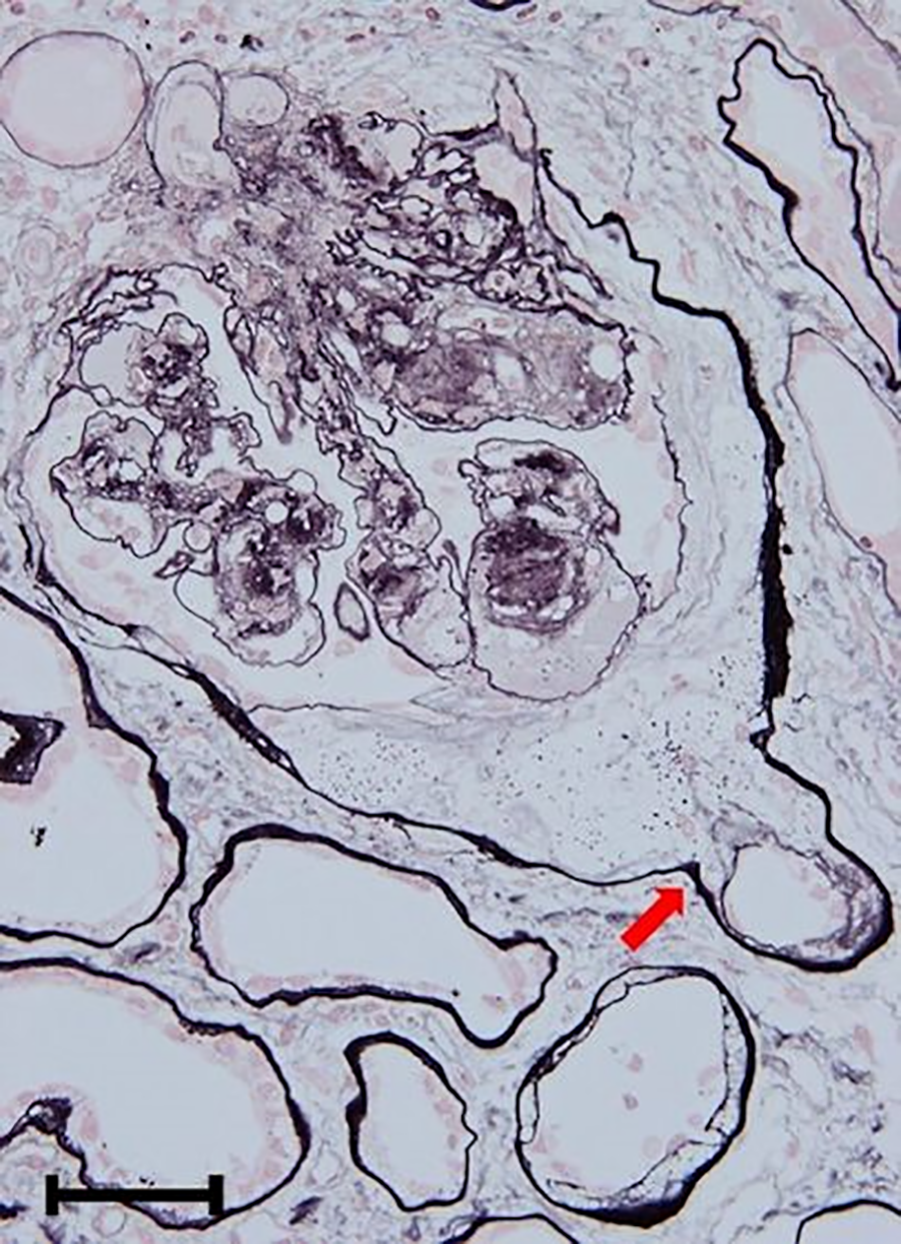
Putative origin of paratubular basement membrane insudative lesions. In most patients, adhesion of the glomerular tuft to the glomerulotubular junction (arrow) was observed, followed by subsequent formation of insudative lesions from the glomerular tubular pole to the proximal convoluted tubule. Original magnification: x 400. Bar = 50 μm. Periodic acid methenamine silver stain.

**Fig 2:**
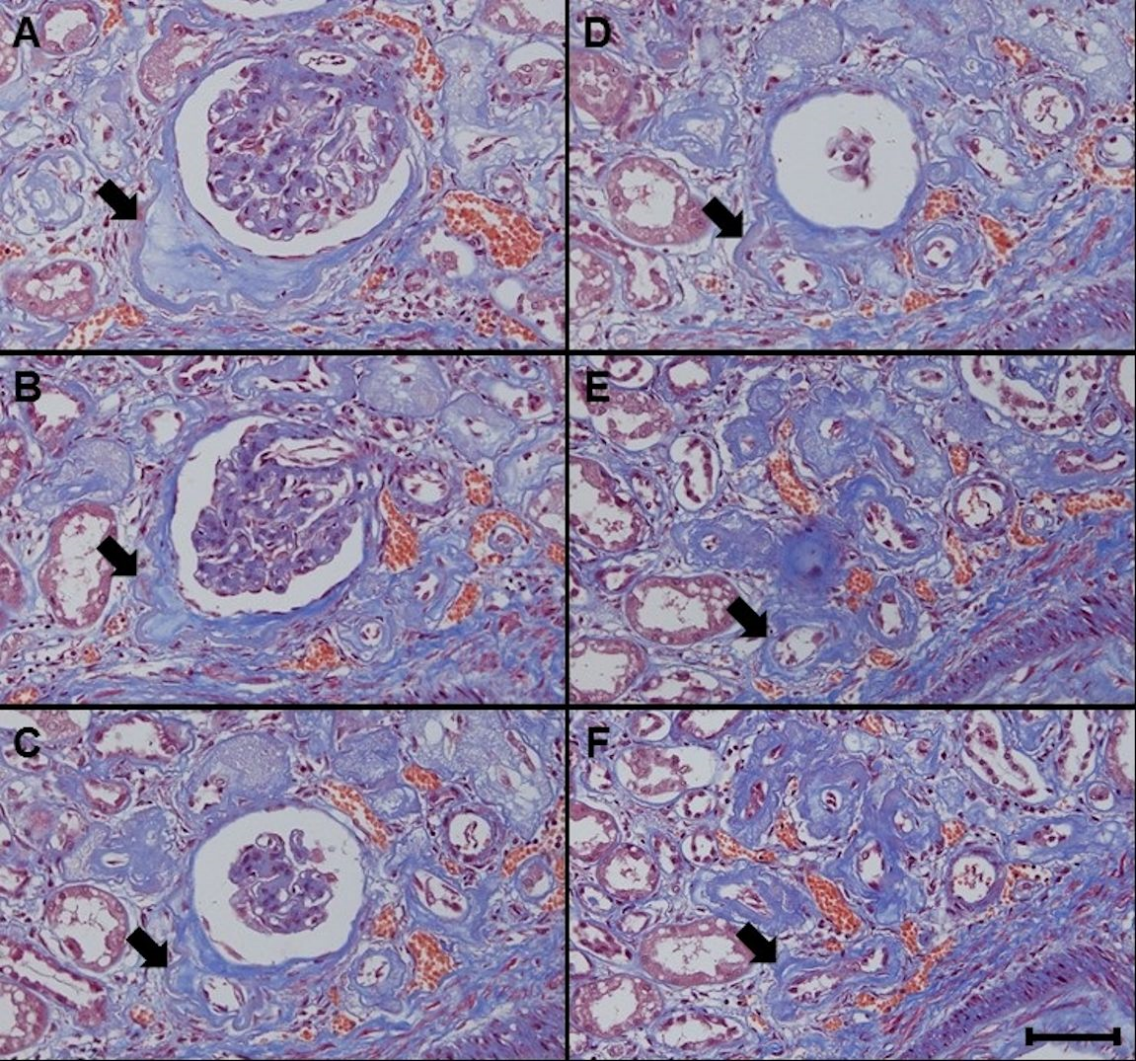
**A-F. Paratubular basement membrane insudative lesions (PTBMIL) extending from the glomerulotubular junction to the proximal convoluted tubule.** Serial sections revealed that PTBMIL (arrows) developed from the abnormal glomerulotubular junction and extended to the proximal convoluted tubule. Original magnification: x 200. Bar = 100 μm. Masson trichrome stain.

**Fig 3:**
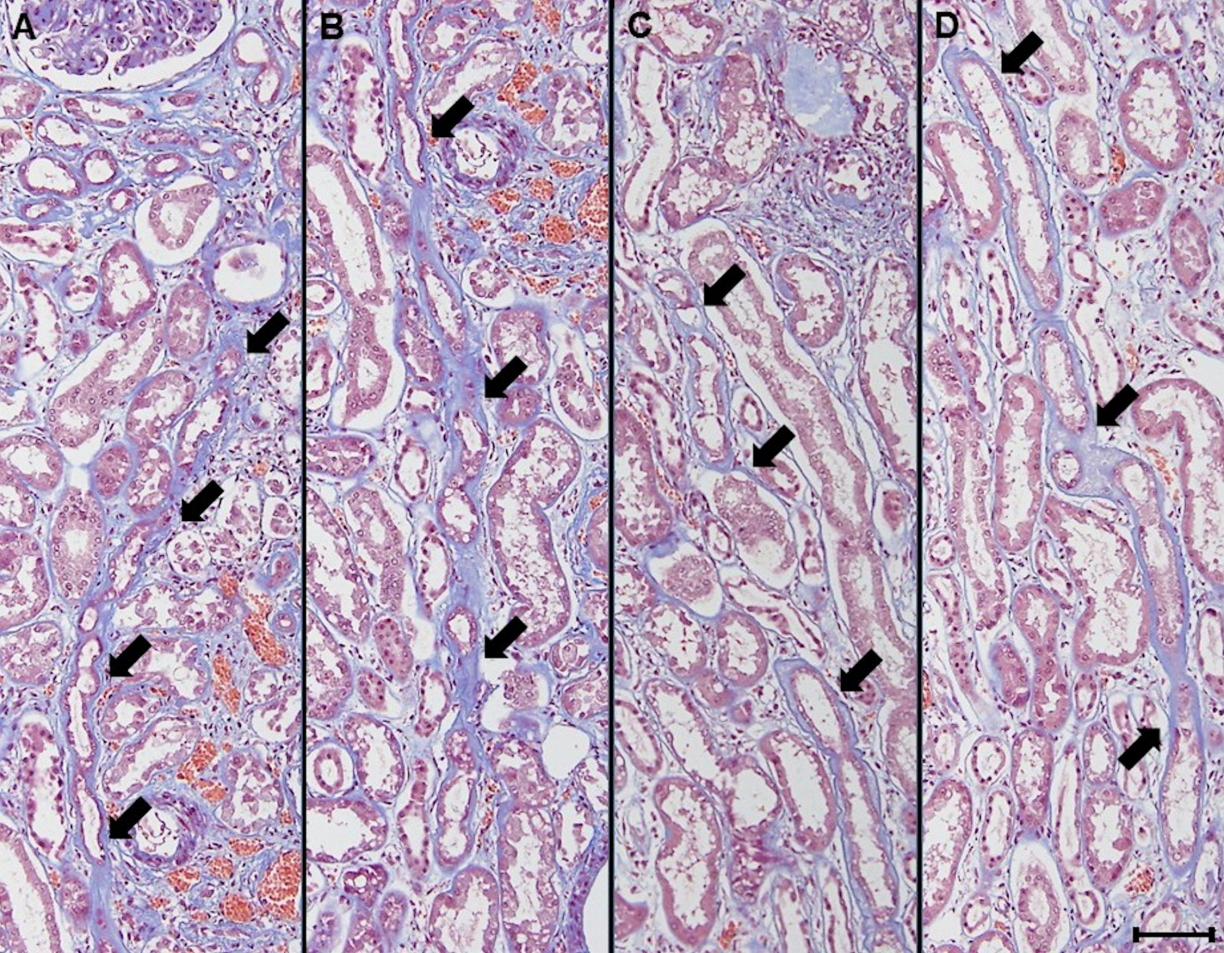
**A-D. Progression of paratubular basement membrane insudative lesions (PTBMIL) from cortex to medulla.** Serial sections revealed that some PTBMIL extended from the glomerular tubular pole in the cortex to the proximal straight tubule in the medulla, and development of PTBMIL paralleled the severity of tubular atrophy. Original magnification: x 200. Bar = 100 μm. Masson trichrome stain.

Of the 142 patients who were screened, 136 met the selection criteria and were enrolled. Among them, 132 patients had varying severity of PTBMIL in the renal cortex and medulla, and the cortical and medullary PTBMIL were similar in extent (S1 Table). In most patients, adhesion of glomerular tufts to the glomerulotubular junction (GTJ) was associated with insudative lesions extending from the glomerular region to the proximal convoluted tubule (Fig 1). Moreover, serial sections revealed that the progression of PTBMIL was varied in tubule, such as from GTJ to proximal convoluted tubule (Fig 2A–2F) and from glomerular tubule in cortex to the proximal straight tubule in medulla (Fig 3A–3D). The overall progression of PTBMIL paralleled the severity of tubular atrophy.

**Fig 4:**
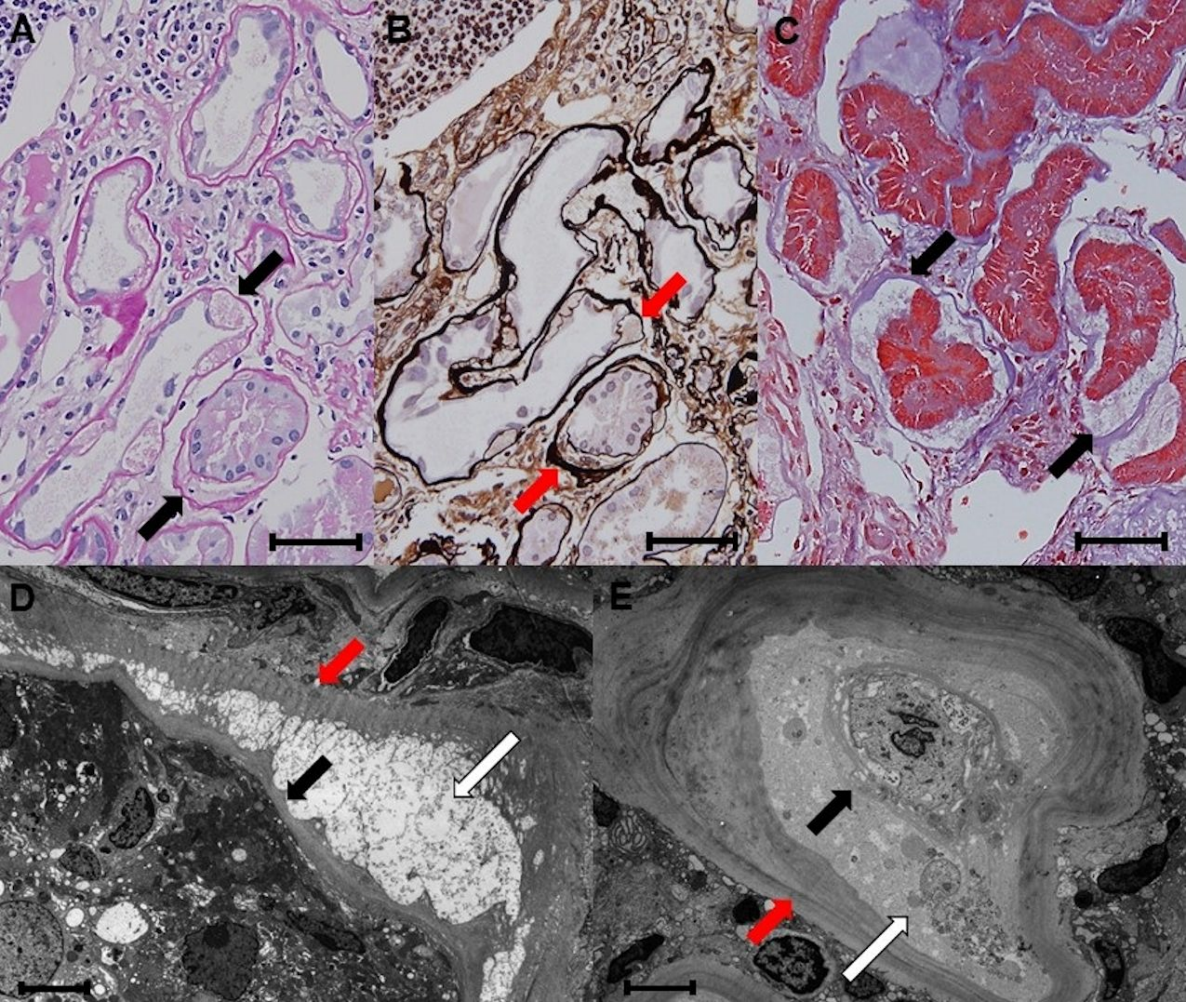
**A-E. Histological features of paratubular basement membrane insudative lesions (PTBMIL).** A-C: Duplication of the tubular basement membrane (TBM) formed by PTBMIL. On periodic acid Schiff (PAS), periodic acid methenamine silver (PAM), and Masson trichrome (MT) stain, duplication of the TBM formed by PTBMIL (arrows) generally coexists with tubular atrophy. A; PAS stain, B; PAM stain, C; MT stain. Original magnification: x 400. Bar = 50μm. D and E: Electron microscopy findings. PTBMIL containing granular and lamellar dense body deposits (white arrows) are located between the thin newly-formed TBM (black arrows) and the thicker primary TBM (red arrows) of the proximal tubule. Original magnification: x 2000, bar = 15μm.

**Fig 5:**
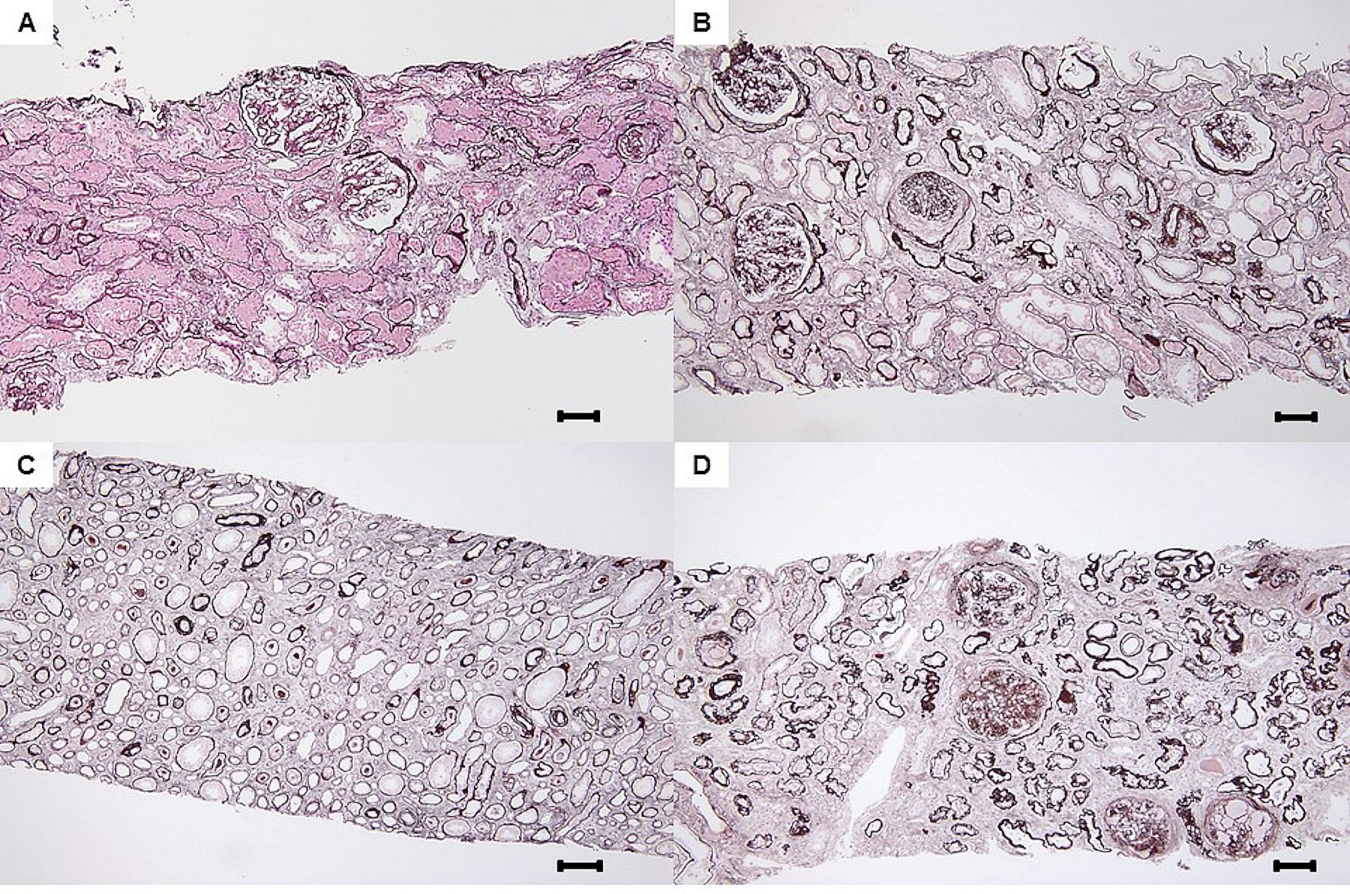
**A-D. Grades of paratubular basement membrane insudative lesions (PTBMIL).** A: Grade 1 in cortex and medulla. PTBMIL was observed in >0% and <25% of tubules of each lesion. Original magnification: x 200. Bar = 100μm, periodic acid methenamine silver (PAM) stain. B and C: Grade 2 in cortex and medulla. PTBMIL was observed in 25–50% of tubules of each lesion. Original magnification of B and C: x 200. Bar = 100μm, PAM stain. D: Grade 3 in cortex and medulla. PTBMIL was observed in >50% of tubules of each lesion. Original magnification of 3D: x 200. Bar = 100μm, PAM stain.

Light microscopic examination with PAS, PAM, and MT staining revealed that duplication of the TBM due to insudative change of the paratubular basement membrane principally coexisted with tubular atrophy (Fig 4A–4C). Duplication of the TBM was seen most clearly with PAM staining. Electron microscopy showed that PTBMIL contained granular and lamellar dense body deposits localized between the thin newly-formed TBM and the thicker primary TBM of the proximal tubule (Fig 4D and 4E). The distribution of PTBMIL in the renal cortex or medulla was classified into 4 categories (none, <25%, 25–50%, and >50%), and the findings are summarized in S1 Table and Fig 5A–5D.

Comparison of histopathological findings among all patients and among groups stratified by the PTBMIL score revealed that glomerular insudative lesions (the presumed origin of PTBMIL) were more frequent in the patients with higher PTBMIL scores and they had higher-grade pathological findings (Table 1 and S2 Table). Correlations of the PTBMIL score and group with other pathologic findings characteristic of DN are also displayed in Table 1. Both the PTBMIL score and the group were significantly correlated with pathological findings of DN, except for the relation between the PTBMIL score and the arteriosclerosis score. The IFTA score was most strongly associated with the PTBMIL score and group (correlation coefficient (*r*) = 0.57 and 0.56, respectively).

**Table 1:** Baseline histopathologic findings in all patients and patients stratified by PTBMIL group, and correlations between PTBMIL score/group and other pathologic findings.

Histopathologic findings					PTBMIL group							Correlation to PTBMIL score	Correlation to PTBMIL group
			All patients		Group 1		Group 2		Group 3		*P* for trend^†^		
					(PTBMIL score 0–2)		(PTBMIL score 3,4)		(PTBMIL score 5,6)				
			(n = 136)		(n = 34)		(n = 50)		(n = 52)			*(r)*	*(r)*
Glomerular insudative lesions (%)			70		47		72		83		<0.01	0.29^‡^	0.29^‡^
Glomerular class	I	(%)	1		3		0		0		<0.001	0.40^‡^	0.38^‡^
	IIA	(%)	19		50		16		2				
	IIB	(%)	26		9		28		37				
	III	(%)	31		32		40		21				
	IV	(%)	23		6		16		40				
Average Glomerular score*			3.6 ± 1.1		2.9 ± 1.1		3.6 ± 1.0		4.0 ± 0.9				
IFTA score	0	(%)	2		9		0		0		<0.001	0.57^‡^	0.56^‡^
	1	(%)	19		59		12		0				
	2	(%)	40		20		56		37				
	3	(%)	39		12		32		63				
Average IFTA score			2.1 ± 0.8		1.3 ± 0.8		2.2 ± 0.6		2.6 ± 0.6				
Interstitial inflammation score	0	(%)	8		29		2		0		<0.01	0.32^‡^	0.27^‡^
	1	(%)	79		62		84		85				
	2	(%)	13		9		14		15				
Average Interstitial inflammation score			1.1 ± 0.5		0.8 ± 0.6		1.1 ± 0.4		1.2 ± 0.4				
Arteriolar hyalinosis score	0	(%)	3		12		0		0		<0.01	0.25^‡^	0.24^‡^
	1	(%)	7		15		2		6				
	2	(%)	90		74		98		94				
Average arteriolar hyalinosis score			1.9 ± 0.4		1.6 ± 0.7		2.0 ± 0.1		1.9 ± 0.2				
Arteriosclerosis score	0	(%)	5	(n = 7)	15	(n = 5)	2	(n = 1)	2	(n = 1)	0.04	0.16	0.17^‡^
	1	(%)	50	(n = 66)	55	(n = 18)	48	(n = 24)	48	(n = 24)			
	2	(%)	45	(n = 60)	30	(n = 10)	50	(n = 25)	50	(n = 25)			
Average arteriosclerosis score			1.4 ± 0.6		1.2 ± 0.7		1.5 ± 0.5		1.5 ± 0.5				

Abbreviations: PTBMIL: paratubular basement membrane insudative lesions, IFTA: interstitial fibrosis and tubular atrophy.

*The glomerular score was defined as follows: Glomerular class I = score 1, class IIA = score 2, class IIB = score 3, class III = score 4, and class IV = score 5.

^†^Tests for linear trend across PTBMIL groups.

^‡^Significant correlation coefficient (*r*).

### Clinical outcome

The median follow-up period was 1.8 years (interquartile range [IQR]: 0.9–3.5 years). During follow-up, the primary endpoint occurred in 104 patients and 5 patients died from causes other than ESRD after refusing dialysis or transplantation.

The characteristics of the 136 patients are listed in Table 2. The age (mean ±SD) at the time of renal biopsy was 61 ± 11 years, and 80% of the patients were men. The mean baseline eGFR was 43.9 ± 22.8 ml/min/1.73 m^2^, while median UP was 2.5 g/day (IQR: 1.5–4.4), and 125 patients (92%) had overt proteinuria. Clinical parameters are also compared in Table 2 among three groups stratified according to the PTBMIL score. Patients in the high PTBMIL score group had a lower BMI, creatinine clearance, eGFR, serum albumin, hemoglobin, and HbA1c levels, as well as higher serum creatinine, UP, and serum uric acid levels. On the other hand, as shown in Table 3, there were no significant differences of mean systolic BP, diastolic BP, and use of ACE-I/ARB among the PTBMIL groups during follow-up among groups stratified by PTBMIL score. In total cohort, the mean systolic BP, diastolic BP, and hemoglobin decreased significantly during follow-up, while mean UP increased significantly versus baseline (Table 3).

**Table 2:** Baseline clinical parameters of all patients and each PTBMIL group.

Clinical parameters		PTBMIL group			
	All patients	Group 1	Group 2	Group 3	*P* for trend^†^		
		(PTBMIL score 0–2)	(PTBMIL score 3,4)	(PTBMIL score 5,6)			
	(n = 136)	(n = 34)	(n = 50)	(n = 52)			
Male (%)	80	74	76	88	0.07
Age (years)	61 ± 11	61 ± 11	63 ± 11	58 ± 11	0.25
BMI (kg/m^2^)	24.0 ± 3.8	25.2 ± 4.1	23.4 ± 3.4	23.8 ± 3.9	0.05
Duration of DM (years)*	14.0 (10.0–20.5)	13.0 (0.5–21.0)	13.5 (10.0–20.0)	16.0 (10.0–23.0)	0.29
SBP (mmHg)	146.9 ± 19.8	142.6 ± 20.4	148.3 ± 18.7	148.5 ± 20.3	0.21
DBP (mmHg)	81.4 ± 13.5	79.4 ± 13.1	79.8 ± 10.8	84.3 ± 15.5	0.17
Retinopathy (%)	71	68	62	83	0.08
Smoker (%)	57	44	62	62	0.14
sCr (mg/dl)*	1.4 (1.0–2.1)	1.0 (0.8–1.2)	1.4 (1.0–1.9)	2.0 (1.4–3.2)	<0.001
CCr (ml/min)	46.9 ± 27.2	67.1 ± 29.2	48.9 ± 24.3	31.5 ± 17.9	<0.001
eGFR (ml/min/1.73m^2^)	43.9 ± 22.8	61.1 ± 20.2	44.4 ± 20.7	32.1 ± 18.9	<0.001
UP (g/day)*	2.5 (1.5–4.4)	1.5 (0.6–2.4)	2.4 (1.7–3.9)	3.7 (2.2–6.5)	<0.001
Normo/Micro/Overt (%)	1/7/92	3/15/82	0/6/94	0/4/96	
Serum albumin (g/dl)	3.1 ± 0.6	3.5 ± 0.5	3.0 ± 0.6	2.8 ± 0.6	<0.001
Hemoglobin (g/dl)	11.9 ± 2.1	13.3 ± 1.9	11.6 ± 1.9	11.2 ± 2.1	<0.001
HbA1c	(%)	7.3 ± 1.6	8.0 ± 1.7	7.1 ± 1.5	7.0 ± 1.5	<0.01
	(mmol/l)	56.0 ± 17.7	64.3 ± 18.4	53.9 ± 16.9	52.7 ± 16.7	
Triglyceride (mg/dl)*	148 (111–206)	157 (124–230)	146 (106–177)	140 (108–206)	0.19
T-Chol (mg/dl)*	202 (171–225)	210 (183–241)	202 (164–221)	200 (168–234)	0.36
Uric acid (mg/dl)	6.7 ± 1.6	6.5 ± 2.1	6.2 ± 1.2	7.2 ± 1.5	0.02
ACE-I or ARB (%)	77	82	74	77	0.62
Antihypertensive agents (n)	2.4 ± 1.4	2.1 ± 1.4	2.4 ± 1.4	2.7 ± 1.5	0.07
OHA therapy (%)	41	47	38	40	0.59
Insulin therapy (%)	45	41	44	48	0.52
ESA (%)	10	9	8	13	0.44

Abbreviations: PTBMIL: paratubular basement membrane insudative lesions, BMI: body mass index, Duration of DM: estimated duration of diabetes mellitus, SBP: systolic blood pressure, DBP: diastolic blood pressure, Retinopathy: diabetic retinopathy, Smoker: current or past smoker, sCr: serum creatinine, CCr: creatinine clearance, eGFR: estimated glomerular filtration rate, UP: urinary protein excretion, Normo/Micro/Overt: normoalbuminuria, microalbuminuria, and overt proteinuria defined as macroalbuminuria or UP > 1g/day, respectively, T-Chol: total cholesterol, ACE-I or ARB: treatment with an angiotensin-converting enzyme inhibitor or angiotensin II type I receptor blocker, respectively, OHA: oral hypoglycemic agent, Insulin therapy: treatment with insulin (including basal-supported oral therapy), ESA: erythropoietin-stimulating agents.

*Median (interquartile range).

^†^Tests for linear trend across PTBMIL groups.

**Table 3:** Comparison of the main clinical parameters between baseline and during follow-up (or at final follow-up) in all patients and among PTBMIL groups.

Clinical parameters		Baseline	During follow-up	*P* Value^†^	PTBMIL group 1	PTBMIL group 2	PTBMIL group 3	*P* Value^‡^
			[1.8 years (0.9–3.5)*]		(PTBMIL score 0–2)	(PTBMIL score 3,4)	(PTBMIL score 5,6)	
					(n = 34)	(n = 50)	(n = 52)	
		(n = 136)	(n = 136)		Parameters during follow-up			
UP (g/day or g/gCr)*		2.5 (1.5–4.4)	3.2 (1.6–5.6)	<0.01	1.4 (0.8–3.1)	3.3 (1.9–5.6)	4.6 (2.5–6.4)	<0.001
SBP (mmHg)		146.9 ± 19.8	141.2 ± 16.1	<0.001	140.7 ± 14.6	141.7 ± 16.7	141.0 ± 16.7	0.95
DBP (mmHg)		81.4 ± 13.5	77.6 ± 10.4	<0.001	76.3 ± 8.4	76.8 ± 8.2	79.3 ± 13.1	0.67
HbA1c	(%)	7.3 ± 1.6	7.2 ± 1.5	0.08	7.9 ± 1.4	7.0 ± 1.4	6.9 ± 1.4	<0.01
	(mmol/l)	56.0 ± 17.7	55.3 ± 16.2		63.0 ± 15.7	53.2 ± 15.6	52.2 ± 15.8	
Hemoglobin (g/dl)		11.9 ± 2.1	11.7 ± 2.0	0.01	12.9 ± 1.8	11.5 ± 1.8	11.0 ± 2.0	<0.001
ACE-I or ARB (%)		77	87	0.12	88	90	83	0.56
		Baseline	At final follow-up		Parameters at final follow-up			
Number of antihypertensive agents		2.4 ± 1.4	2.9 ± 1.4	<0.001	2.8 ± 1.4	3.1 ± 1.4	2.9 ± 1.4	0.58
OHA therapy (%)		41	34	0.21	38	36	29	0.61
Insulin therapy (%)		45	53	0.18	53	50	56	0.84
ESA (%)		10	30	<0.001	12	26	46	<0.01
Outcome								
Primary outcome (%)			78 (n = 106)		53	86	87	<0.001
Death (%)			3 (n = 4)		0	4	4	0.68

Abbreviations: PTBMIL: paratubular basement membrane insudative lesions, UP: urinary protein excretion, SBP: systolic blood pressure, DBP: diastolic blood pressure, ACE-I or ARB: treatment with an angiotensin-converting enzyme inhibitor or angiotensin II type I receptor blocker, respectively, OHA: oral hypoglycemic agent, Insulin therapy: treatment with insulin (including basal-supported oral therapy), ESA: erythropoietin-stimulating agents, Primary outcome: initiation of dialysis because of end-stage renal disease or ≥40% decline of the estimated glomerular filtration rate. Parameters during follow-up are average annual parameters. Baseline UP was measured in a 24-hour urine specimen (g/day), whereas UP (g/gCr) in spot urine samples was employed if UP (g/day) was not available during follow-up. Use of ACE-I or ARB during follow-up was defined as treatment with the relevant drug for more than half of the follow-up period.

*Median (interquartile range).

^†^Categorical variables were analyzed with the two-group proportion test, while continuous variables were compared by using the paired t-test.

^‡^Categorical variables were analyzed with the chi-square test or Fisher’s exact test, and continuous variables were compared by using ANOVA or Kruskal-Wallis H test.

**Fig 6:**
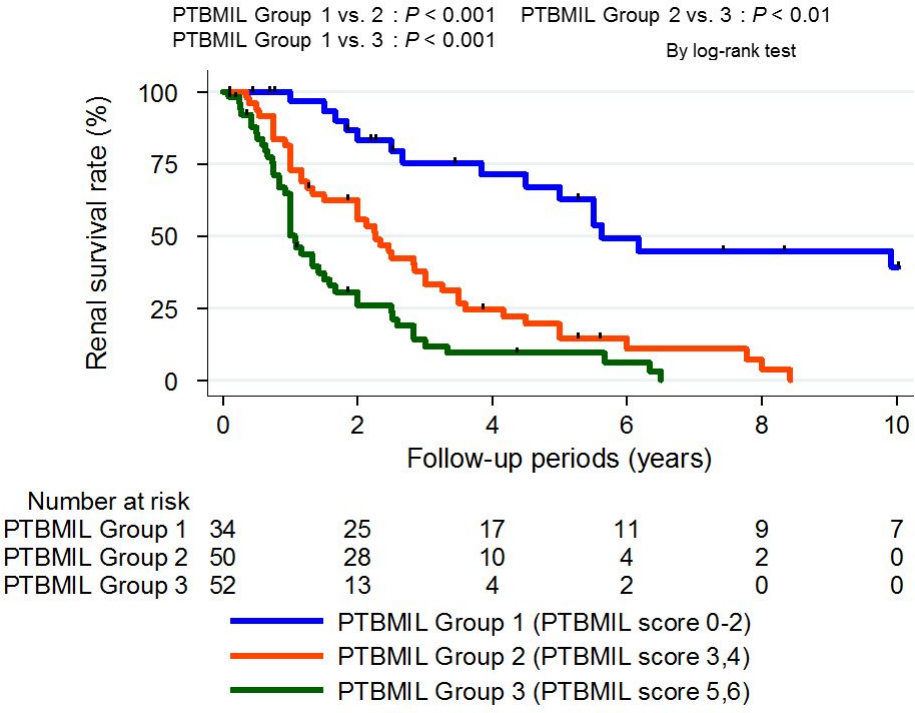
Renal survival rate stratified by PTBMIL group. The estimated 2-year renal survival rate was 83% in PTBMIL group 1, 56% in PTBMIL group 2, and 26% in PTBMIL group 3. There were significant differences of the renal survival rate between the different PTBMIL groups. Outcome: ≥40% decline of estimated glomerular filtration rate or dialysis due to end-stage renal disease. The log-rank test was used for survival analysis. Abbreviations: PTBMIL: paratubular basement membrane insudative lesions.

Kaplan-Meier curves for renal survival stratified according to PTBMIL group are displayed in Fig 6. The high PTBMIL score group had a significantly lower renal survival rate than the other groups, and there were significant differences of renal survival between the PTBMIL groups (P<0.01).

Cox proportional hazards analysis revealed that the PTBMIL score was positively correlated with the renal endpoint in categorical analyses after adjustment for various known prognostic indicators, such as the duration of diabetes, diabetic retinopathy, baseline systolic BP, UP, and eGFR. An increase of the PTBMIL score by 1 point was associated with a significantly higher risk of the renal endpoint (HR: 1.28, 95% CI: 1.08–1.51). When the PTBMIL score was incorporated as a categorical variable, the HR for the endpoint generally increased along with the PTBMIL score (S3 Table). Based on these results, the patients were classified into three PTBMIL groups: group 1 (PTBMIL score 0–2), group 2 (PTBMIL score 3–4), and group 3 (PTBMIL score 5–6). As was seen with the PTBMIL score, an increase of the PTBMIL group from 1 to 3 was associated with higher HRs for the endpoint. Compared with PTBMIL group 1, the HRs for the outcome of PTBMIL groups 2 and 3 were 2.32 (1.20–4.51) and 3.12 (1.48–6.58), respectively (Table 4). The difference of Harrell’s c-index between Cox regression models with or without the PTBMIL group/PTBMIL score is also shown in Table 4. Adding the PTBMIL group and PTBMIL score to a multivariate model with the same covariates as model 2 resulted in a significantly higher c-index (PTBMIL group: increase of 0.02 [0.00–0.04]; PTBMIL score: increase of 0.02, 95%CI [0.00–0.05]).

**Table 4:** Univariate and multivariate Cox proportional hazard models incorporating PTBMIL group and Harrell’s C-index of models with or without the PTBMIL group/PTBMIL score.

PTBMIL Group	≥40% Decline of eGFR or Dialysis Hazard Ratio (95% CI)			
	Univariate	Model 1		Model 2
Group 1 (PTBMIL score 0–2)	Reference	Reference		Reference
Group 2 (PTBMIL score 3, 4)	3.79 (2.04–7.05)	3.45 (1.82–6.52)		2.32 (1.20–4.51)
Group 3 (PTBMIL score 5, 6)	6.77 (3.60–12.73)	6.77 (3.49–13.14)		3.12 (1.48–6.58)
Cox Regression Model	C-index (95% CI)		Difference of C-index (95% CI)	
Model with covariate only	0.76 (0.71–0.80)			
Model with PTBMIL group	0.77 (0.73–0.82)		0.02 (0.00–0.04)	
Model with PTBMIL score	0.78 (0.74–0.82)		0.02 (0.00–0.05)	

Model 1: Adjusted for age, gender, body mass index, estimated duration of diabetes mellitus, diabetic retinopathy, and systolic blood pressure at baseline. Model 2: Adjusted for the covariates in model 1, log converted urinary protein excretion, and estimated glomerular filtration rate at baseline. Covariates: age, gender, body mass index, estimated duration of diabetes mellitus, diabetic retinopathy, and systolic blood pressure, estimated glomerular filtration rate, and log (urinary protein excretion) at baseline. In the multivariate Cox regression analyses with PTBMIL group or score to calculate c-index, both PTBMIL group and score are employed as categorical variables. Abbreviations: PTBMIL: paratubular basement membrane insudative lesions, c-index: concordance index, 95% CI: 95% confidence interval.

### Relation of the IFTA score with PTBMIL and other factors

Because we found a close relationship between the IFTA score and the PTBMIL score, we investigated the pathological findings closely related to tubulointerstitial injury. As shown in S4 Table, the IFTA score was not only strongly correlated with the PTBMIL score but also with glomerular insudative lesions (*r* = 0.57 and 0.40, respectively), while the glomerular class showed the strongest correlation with the IFTA score (*r =* 0.63). There were differences in the strength of the correlation of the glomerular class and the arteriosclerosis score with the IFTA score or the PTBMIL score (S4 Table; correlation of glomerular class with the IFTA score and PTBMIL score: *r* = 0.63 and 0.40, respectively; correlation of the arteriosclerosis score with the IFTA score and PTBMIL score: *r* = 0.32 and 0.16, respectively).

Next, to further investigate the association of PTBMIL with the pathogenesis of interstitial injury in DN, we classified the patients into 4 categories according to their IFTA scores. The distribution of PTBMIL groups in each IFTA score category and the renal prognosis of the patients in each PTBMIL group were investigated. In addition, factors related to hypertension and arteriosclerosis were compared among patients from the different IFTA score categories within each PTBMIL group.

The 4 patients with an IFTA score of 0 all belonged to PTBMIL group 1. Among patients with IFTA score of 1, there was a significant difference of the renal survival rate between PTBMIL groups 1 and 2 (P <0.01) (S1 Fig). However, among patients with an IFTA score of 2 or 3, there were no significant differences of the renal survival rate among the PTBMIL groups (S1 Fig). Intriguingly, the patients with higher IFTA scores in each PTBMIL group had a higher systolic BP, a higher arteriosclerosis score, and used more antihypertensive agents, although the differences among some groups were not significant (S5 Table).

## Discussion

It has been reported that tubular changes, such as TBM thickening and duplication, may occur subsequent to alterations in the selectivity of glomerular permeability, and these changes are more frequent in advanced DN than in other renal diseases associated with tubular atrophy of similar severity [21,22]. Najafian *et al*. [23] reported that GTJ abnormalities, particularly adhesion of the glomerular tuft to the GTJ, were often observed in advanced DN, in parallel with TBM duplication and atrophy of the connecting proximal tubules. They speculated that GTJ abnormalities may lead to impaired filtration and development of insudative lesions extending from Bowman’s space to the proximal tubule [23,24]. These abnormalities are similar to the tip lesions of focal segmental glomerulosclerosis (FSGS). KRIZ *et al*. [25] demonstrated that misdirected filtration and spreading of filtrate into the space between the parietal epithelium and parietal basement membrane led to obstruction and subsequent tubular atrophy in a rat models of FSGS. Based on these reports, it is possible that PTBMIL result from aberrant progression of insudative lesions (from Bowman’s space to the proximal tubule) due to GTJ abnormalities secondary to glomerular insudative lesions.

GTJ abnormalities were frequent in the present study population and PTBMIL varied in severity, while glomerular insudative lesions showed a higher prevalence in the higher PTBMIL groups (Table 1) that was consistent with the mechanism proposed above for the origin and progression of PTBMIL. Although we did not observe any glomerular insudative lesions in a few of the patients with moderate PTBML (Group 2) and severe PTBMIL (Group 3), this might have been due to much more extensive global glomerulosclerosis in these higher PTBMIL groups and sampling bias during renal biopsy. This idea is partly supported by the finding that patients without glomerular insudative lesions had fewer glomeruli without global glomerulosclerosis than patients with these lesions in each PTBMIL group (S2 Table). On the other hand, the PTBMIL score and group were most strongly associated with the IFTA score among all pathologic parameters (*r* = 0.57 and 0.56, respectively), suggesting a close relationship between PTBMIL and tubulointerstitial injury. In addition, we observed that progression of PTBMIL paralleled the severity of tubular atrophy (Figs 2–5), in agreement with the results of previous studies [23,24]. Based on these findings, PTBMIL could be the main cause of tubulointerstitial damage in DN, especially tubular atrophy.

Furthermore, PTBMIL were strongly associated with the renal prognosis, and added significant prognostic value to known indicators of renal progression. Since the IFTA score has incremental predictive power for renal progression in addition to known indicators [15], it seems reasonable for PTBMIL (the causative lesions of IFTA) to be a useful prognostic indicator for DN.

Another cause of tubular atrophy in DN is ischemia due to nephrosclerosis, especially hypertensive nephrosclerosis. When ischemia results in tubular atrophy, the atrophic tubules generally do not have TBM duplication or thickening. Therefore, we can determine the predominant pathogenesis of tubular atrophy, paratubular basement membrane insudative changes in DN or ischemia in hypertensive nephrosclerosis, by comparing the PTBMIL score with the IFTA score. If the IFTA score is high despite a relatively low PTBMIL score, interstitial lesions might be mainly related to nephrosclerosis rather than DN. The results of our comparison of blood pressure and the severity of arteriosclerosis between patients in the same PTBMIL group across IFTA score categories might support this speculation (S5 Table). Differences in the strength of the correlation between the glomerular class or arteriosclerosis score and the IFTA score or PTBMIL score were also consistent with this concept (S4 Table). There was a significant difference of the renal survival rate among the PTBMIL groups in patients with an IFTA score of 1, but not in patients with IFTA scores of 2 or 3. Thus, differences in the pathogenesis of IFTA might be more important in the early stage of tubulointerstitial lesions than in the advanced stage. Confirming the clinical importance of these lesions in early DN could lead to further investigation of the putative pathogenesis of PTBMIL.

The main limitations of this study were its retrospective cohort design and performance at a single center with insufficiently standardized indications for renal biopsy, suggesting that selection bias could have occurred. Another limitation is that the management of DN after renal biopsy was not adequately examined or adjusted in this study. However, comparisons of clinical parameters during follow-up may suggest that there were no obvious major differences of current standard treatment factors of DN (Table 3).

In conclusion, varying levels of PTBMIL were observed in our patients with advanced DN. The PTBMIL score and PTBMIL group were both significantly associated with the renal prognosis independently of known prognostic indicators of DN. Moreover, adding the PTBMIL score or group to the known prognostic factors significantly improved prediction of the renal outcome. Accordingly, investigation of PTBMIL may be useful for predicting the renal prognosis of patients with biopsy-proven DN. However, further investigation will be required to validate our results, especially in patients with early DN.

## Supporting information

S1 Table Distribution of cortical and medullary PTBMIL grades. (PDF)

S2 Table Association between glomerular insudative lesions and the number of glomeruli without global glomerulosclerosis in each PTBMIL group. (PDF)

S3 Table Univariate and multivariate Cox proportional hazard models incorporating PTBMIL score. (PDF)

S4 Table Correlation coefficients of IFTA score and PTBMIL score with other pathologic findings. (PDF)

S5 Table Differences of the systolic blood pressure, number of antihypertensive drug, and arteriosclerosis score among patients stratified according to IFTA scores in each PTBMIL group. (PDF)

S1 Fig Renal survival curves of patients in each PTBMIL group stratified according to IFTA score. A: Renal survival rate of PTBMIL groups in patients with IFTA score 1. B: Renal survival rate of PTBMIL groups in patients with IFTA score 2. C: Renal survival rate of PTBMIL groups in patients with IFTA score 3. In patients with IFTA score 1, there was a significant renal survival rate between PTBMIL group 1 and PTBMIL group 2 (P = <0.01). However, in both groups of patients with IFTA score 2 and score 3, there was not significant trend of renal survival rate among PTBMIL groups.
(PDF)
