# Supplementary material for: Paratubular basement membrane insudative lesions predict renal prognosis in patients with type 2 diabetes and biopsy-proven diabetic nephropathy
Source: PLoS One. 2017 Aug 15;12(8):e0183190. doi: 10.1371/journal.pone.0183190 (PMC5557586; doi:10.1371/journal.pone.0183190)
Supplement: S1 Table — (PDF) [file pone.0183190.s001.pdf]

S1 Table 1. Distribution of cortical and medullary PTBMIL grades.

	Cortex	Medulla	Cortex + Medulla	
Grade 0 (Absence of PTBMIL)	10 (7)	7 (5)	PTBMIL score 0	4 (3)
Grade 1 (PTBMIL <25%)	27 (20)	41 (30)	PTBMIL score 1	9 (7)
Grade 2 (PTBMIL: 25-50%)	57 (42)	50 (37)	PTBMIL score 2	21 (15)
Grade 3 (PTBMIL >50%)	42 (31)	38 (28)	PTBMIL score 3	15 (11)
			PTBMIL score 4	35 (26)
			PTBMIL score 5	26 (19)
			PTBMIL score 6	26 (19)

Number (%)

Abbreviations

PTBMIL: paratubular basement membrane insudative lesions
