# Supplementary material for: Paratubular basement membrane insudative lesions predict renal prognosis in patients with type 2 diabetes and biopsy-proven diabetic nephropathy
Source: PLoS One. 2017 Aug 15;12(8):e0183190. doi: 10.1371/journal.pone.0183190 (PMC5557586; doi:10.1371/journal.pone.0183190)
Supplement: S2 Table — (PDF) [file pone.0183190.s002.pdf]

S2 Table. The association between the presence of glomerular insudative lesions and number of glomeruli without global glomerulosclerosis in patients stratified by PTBMIL groups.

Histopathologic findings		All patients (n=136)	PTBMIL group: Group 1 (PTBMIL score 0-2) (n=34)		Group 2 (PTBMIL score 3,4) (n=50)		Group 3 (PTBMIL score 5,6) (n=52)		*P* for trend†
Glomerular insudative lesions	(%)	70	47		72		83		<0.01
	(- or +)		(-)	(+)	(-)	(+)	(-)	(+)	
Number of glomeruli* without global glomerulosclerosis			9 (6-13)	12 (5-22)	11 (10-13)	15 (9-21)	8 (4-12)	10 (5-16)	
Number of glomeruli*		18 (12-28)	14 (8-20)		19 (13-28)		21 (15-29)		<0.01

Abbreviations

PTBMIL: paratubular basement membrane insudative lesions, Number of glomeruli: mean number of glomeruli obtained by renal biopsy. *Median (interquartile range). †Tests for linear trend across PTBMIL groups.

Number of glomeruli without global glomerulosclerosis was larger in groups with glomerular insudative lesions than in groups without those in each PTBMIL group, whereas it was not statistically significant.
