# Supplementary material for: Paratubular basement membrane insudative lesions predict renal prognosis in patients with type 2 diabetes and biopsy-proven diabetic nephropathy
Source: PLoS One. 2017 Aug 15;12(8):e0183190. doi: 10.1371/journal.pone.0183190 (PMC5557586; doi:10.1371/journal.pone.0183190)
Supplement: S3 Table — (PDF) [file pone.0183190.s003.pdf]

S3 Table. Univariate and multivariate Cox proportional hazard models incorporating PTBMIL score.

PTBMI score	≥40% Decline of eGFR or Dialysis Hazard Ratio (95% CI)		
	Univariate	Model 1	Model 2
PTBMI score 0 (n=4)	0.22 (0.03 - 1.80)	0.32 (0.04 - 2.78)	0.88 (0.10 - 7.97)
PTBMI score 1 (n=9)	0.54 (0.17 - 1.68)	0.54 (0.17 - 1.69)	0.51 (0.15 - 1.71)
PTBMI score 2 (n=21)	Reference	Reference	Reference
PTBMI score 3 (n=15)	3.78 (1.68 - 8.48)	3.97 (1.73 - 9.08)	2.73 (1.17 - 6.39)
PTBMI score 4 (n=35)	2.51 (1.22 - 5.14)	2.30 (1.09 - 4.88)	1.67 (0.77 - 3.59)
PTBMI score 5 (n=26)	4.01 (1.86 - 8.63)	4.51 (2.01 - 10.10)	2.27 (0.96 - 5.35)
PTBMI score 6 (n=26)	6.56 (3.11 - 13.85)	6.40 (2.91 - 14.07)	3.21 (1.40 - 7.38)
Per PTBMI score	1.54 (1.34 - 1.76)	1.52 (1.32 - 1.76)	1.28 (1.08 - 1.51)

Model 1: Adjusted for age, gender, body mass index, estimated duration of diabetes mellitus, diabetic retinopathy, and systolic blood pressure at baseline

Model 2: Adjusted for the covariates in model 1, log converted urinary protein excretion, and estimated glomerular filtration rate at baseline

Covariates: age, gender, body mass index, estimated duration of diabetes mellitus, diabetic retinopathy, and systolic blood pressure, estimated glomerular filtration rate, and log (urinary protein excretion) at baseline. In the multivariate Cox regression analyses with PTBMIL score to calculate c-index, PTBMIL score is employed as categorical variables.

Abbreviations

PTBMIL: paratubular basement membrane insudative lesions, c-index: concordance index, 95% CI: 95% confidence interval.
