# Supplementary material for: Paratubular basement membrane insudative lesions predict renal prognosis in patients with type 2 diabetes and biopsy-proven diabetic nephropathy
Source: PLoS One. 2017 Aug 15;12(8):e0183190. doi: 10.1371/journal.pone.0183190 (PMC5557586; doi:10.1371/journal.pone.0183190)
Supplement: S4 Table — (PDF) [file pone.0183190.s004.pdf]

S4 Table. Correlation coefficients of IFTA score and PTBMIL score with other pathologic findings.

	IFTA score	PTBMIL score	Glomerular insudative lesion	Glomerular class	Interstitial inflammation score	Arteriolar hyalinosis score	Arteriosclerosis score
IFTA score	-	0.57*	0.40*	0.63*	0.33*	0.31*	0.32* (n=133)
PTBMIL score	-	-	0.29*	0.40*	0.32*	0.25*	0.16 (n=133)

Abbreviations

IFTA: interstitial fibrosis and tubular atrophy, PTBMIL: paratubular basement membrane insudative lesions.

*Significant correlation coefficient (*r*)
