# Supplementary material for: Paratubular basement membrane insudative lesions predict renal prognosis in patients with type 2 diabetes and biopsy-proven diabetic nephropathy
Source: PLoS One. 2017 Aug 15;12(8):e0183190. doi: 10.1371/journal.pone.0183190 (PMC5557586; doi:10.1371/journal.pone.0183190)
Supplement: S5 Table — (PDF) [file pone.0183190.s005.pdf]

S5 Table. Difference of systolic blood pressure, number of antihypertensive drug, and arteriosclerosis score among groups stratified according to IFTA scores in each PTBMIL group.

PTBMIL group 1 (n=34)	IFTA score 0 (n=3)	IFTA score 1 (n=20)	IFTA score 2 (n=7)	IFTA score 3 (n=4)	*P*-for trend*
Systolic blood pressure (mmHg)	124 ± 16	141.6 ± 19.1	144.9 ± 15.0	158.0 ± 30.8	0.11
Number of antihypertensive drug (n)	1.3 ± 1.5	2.1 ± 1.6	1.9 ± 0.9	3.0 ± 1.2	0.20
Average arteriosclerosis score	1.0 ± 0.8	1.1 ± 0.7	1.1 ± 0.7	1.7 ± 0.6	0.22
PTBMIL group 2 (n=50)	IFTA score 0 (n=0)	IFTA score 1 (n=6)	IFTA score 2 (n=28)	IFTA score 3 (n=16)	*P* for trend*
Systolic blood pressure (mmHg)		136.8 ± 13.8	148.6 ± 18.3	152.1 ± 20.4	0.11
Number of antihypertensive drug (n)		1.8 ± 1.2	2.0 ± 1.3	3.2 ± 1.4	0.02
Average arteriosclerosis score		1.0 ± 0.6	1.5 ± 0.5	1.7 ± 0.5	0.02
PTBMIL group 3 (n=52)	IFTA score 0 (n=0)	IFTA score 1 (n=0)	IFTA score 2 (n=19)	IFTA score 3 (n=33)	*P*-value†
Systolic blood pressure (mmHg)			141.7 ± 19.7	152.4 ± 19.9	0.08
Number of antihypertensive drug (n)			2.1 ± 1.4	3.1 ± 1.4	0.02
Average arteriosclerosis score			1.3 ± 0.6	1.6 ± 0.5	0.06

Abbriviation

IFTA: interstitial fibrosis and tubular atrophy, PTBMIL: paratubular basement membrane insudative lesions

[*]Tests for linear trend across IFTA scores. [†]P-value was calculated by Wilcoxon signed rank test.
