# Supplementary material for: Paratubular basement membrane insudative lesions predict renal prognosis in patients with type 2 diabetes and biopsy-proven diabetic nephropathy
Source: PLoS One. 2017 Aug 15;12(8):e0183190. doi: 10.1371/journal.pone.0183190 (PMC5557586; doi:10.1371/journal.pone.0183190)
Supplement: S1 Fig — A: Renal survival rate of PTBMIL groups in patients with IFTA score 1. B: Renal survival rate of PTBMIL groups in patients with IFTA score 2. C: Renal survival rate of PTBMIL groups in patients with IFTA score 3. In patients with IFTA score 1, there was a significant renal survival rate between PTBMIL group 1 and PTBMIL group 2 (P = <0.01). However, in both groups of patients with IFTA score 2 and score 3, there was not significant trend of renal survival rate among PTBMIL groups. (PDF) [file pone.0183190.s006.pdf]

S1 Fig A

Patients with IFTA score 1

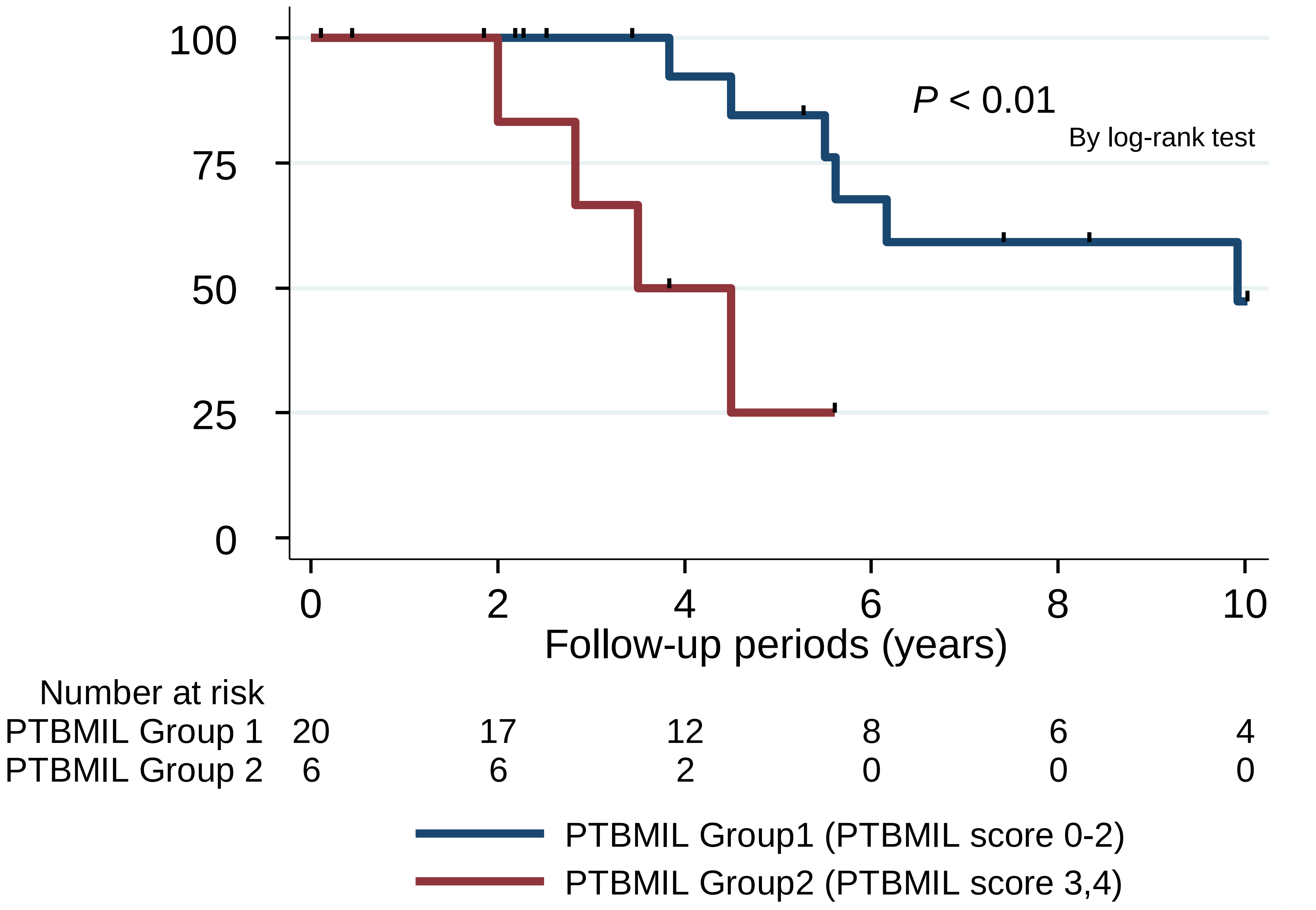

S1 Fig B

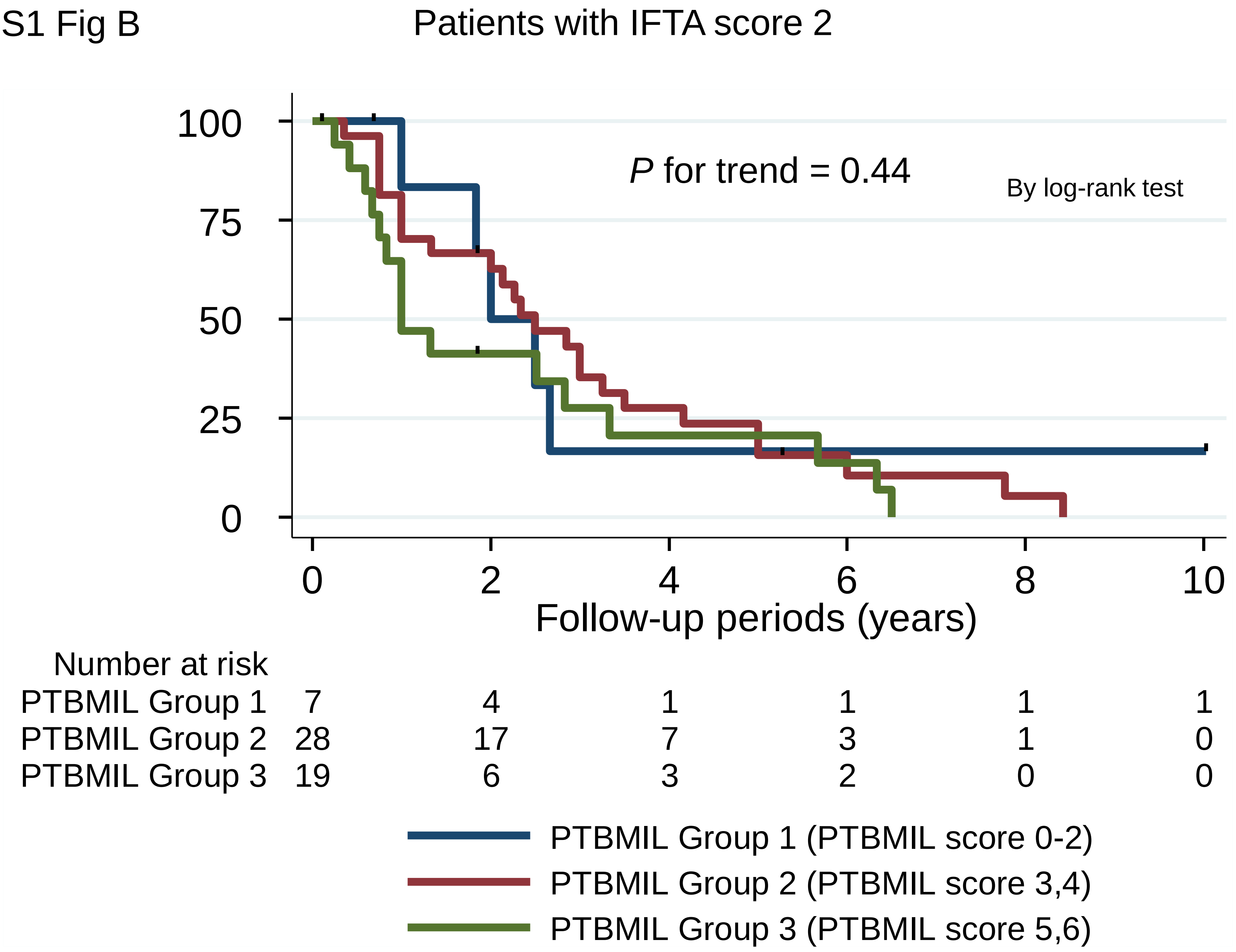
Patients with IFTA score 2
100
75
50
25
0
P for trend = 0.44
By log-rank test
0
2
4
6
8
10
Follow-up periods (years)
Number at risk
PTBMIL Group 1 7 4 1 1 1 1
PTBMIL Group 2 28 17 7 3 1 0
PTBMIL Group 3 19 6 3 2 0 0
PTBMIL Group 1 (PTBMIL score 0-2)
PTBMIL Group 2 (PTBMIL score 3,4)
PTBMIL Group 3 (PTBMIL score 5,6)

S1 Fig C

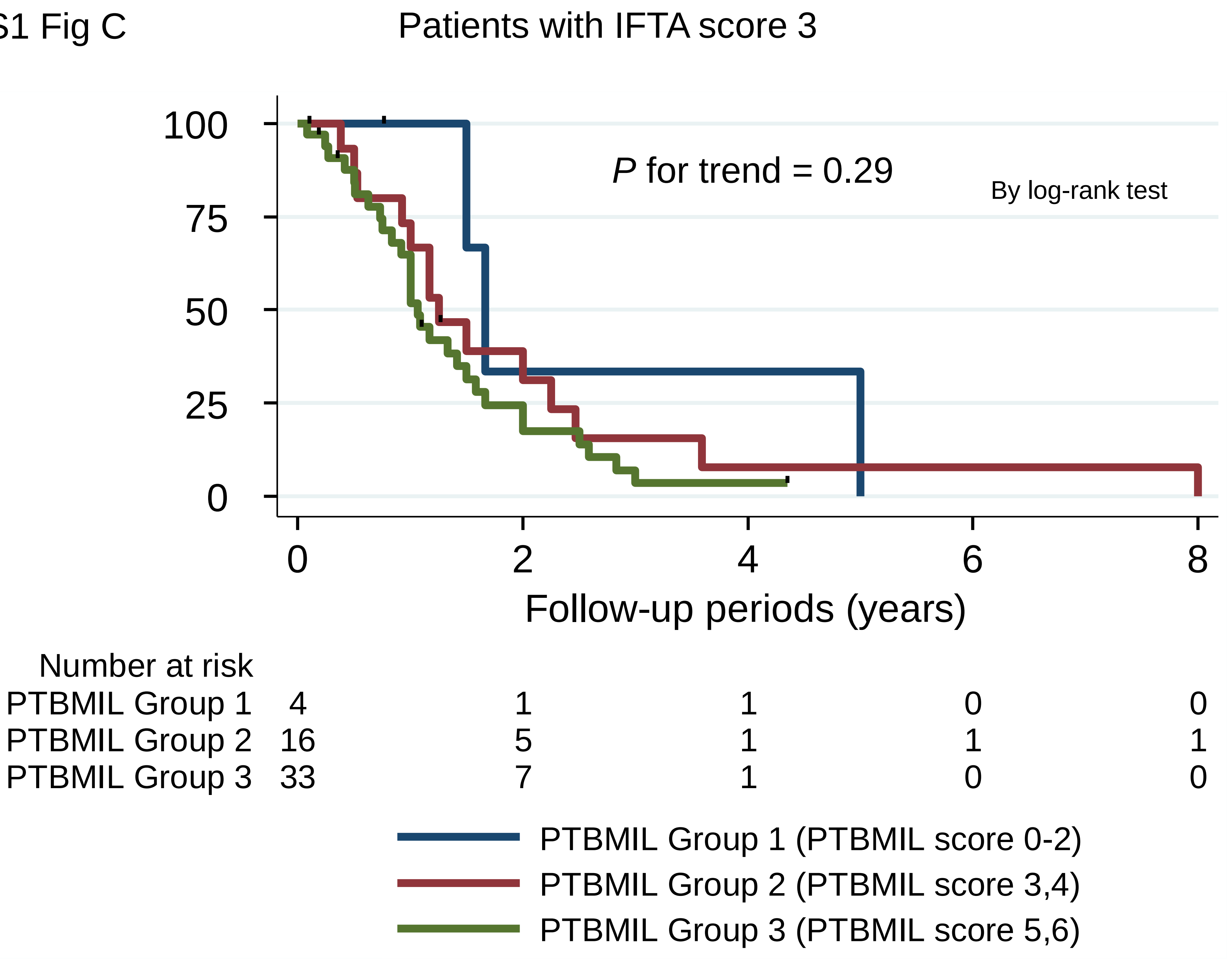

Patients with IFTA score 3
100
75
50
25
0
P for trend = 0.29
By log-rank test
0
2
4
6
8
Follow-up periods (years)
Number at risk
PTBMIL Group 1 4 1 1 0 0
PTBMIL Group 2 16 5 1 1 1
PTBMIL Group 3 33 7 1 0 0
PTBMIL Group 1 (PTBMIL score 0-2)
PTBMIL Group 2 (PTBMIL score 3,4)
PTBMIL Group 3 (PTBMIL score 5,6)
